# Prefemoral Fat Pad Impingement Syndrome: Correlation between Ultrasonography and Magnetic Resonance Imaging

**DOI:** 10.5334/jbsr.3606

**Published:** 2024-06-07

**Authors:** Sophie Dheur, Felicie Lorthioir

**Affiliations:** 1 Intern in Radiology, Radiology Department, CHU de Liège, Belgium; 2 Consultant radiologist, CHU de Liège, Belgium

**Keywords:** Knee, prefemoral fat pad, impingement syndrome

## Abstract

*Teaching point:* Prefemoral fat pad impingement syndrome is one of the fat pad impingements of the knee and can be assessed with ultrasonography.

## Case History

A 12-year-old girl was admitted to our emergency department with nontraumatic right knee pain. The little girl complained of severe restrictions on extensions.

**Figure 1:**
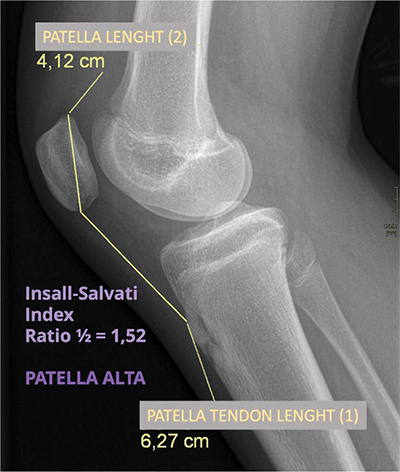
Lateral radiograph of the knee. Joint effusion and patella alta.

Simple knee radiographs showed knee joint effusion and a patella alta defined by the Insall-Salvati index calculated to 1.52 (Figure 1). No traumatic or suspect lesion was detected.

**Figure 2:**
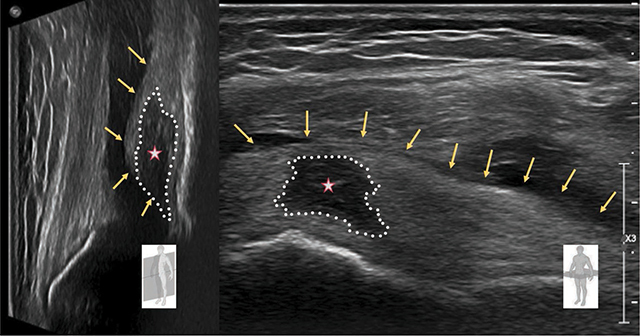
Ultrasonography of the knee. Joint effusion and hypoechoic edema with enlargement of the prefemoral fat pad (asterisk).

An ultrasonography (US) (Figure 2) was performed and confirmed a significant joint effusion and showed hypoechoic edema with enlargement of the prefemoral fat pad (PFP) (asterisk).

In this context, magnetic resonance imaging (MRI) was performed, and all the abnormalities cited above were confirmed with a very good correlation between US and MRI.

**Figure 3:**
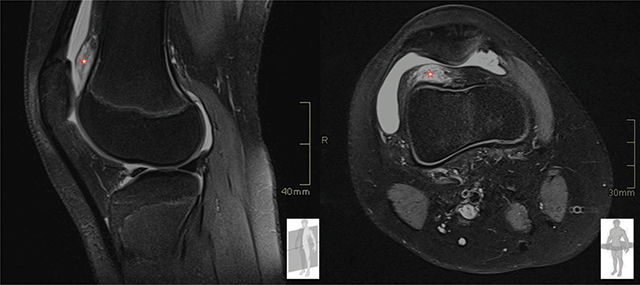
MRI, T2-weighter fat suppressed. PFP edema with mass-like protrusion into the suprapatellar pouch. Joint effusion.

T2-weighted fat-suppressed MR images (Figure 3) demonstrate PFP edema, with scarring and mass-like protrusion into the suprapatellar pouch (asterisk) associated with joint effusion.

## Comments

The PFP is one of the three anterior knee fat pads, along with the infrapatellar and quadriceps fat pads. PFP is located anterior to the distal femur metaphysis and posteriori to the suprapatellar recess.

Clinically, prefemoral fat pad impingement syndrome (PFPIS) includes anterior knee pain exacerbated by hyperextension.

Depending on the clinical presentation, the differential diagnosis is large, including, among other things, osteochondral lesion, meniscal tear, patellar dislocation, and quadriceps tendonitis.

According to the literature, PFPIS is due in part to patellar movement.

In complete extension, the patella is located on the proximal side of the femoral trochlea and doesn’t get in touch with the cortical trochlea. During flexion, the patella moves from the proximal to distal direction along trochlear groove. During this shift, the PFP could be through to “sandwich” [1].

In cases of morphological abnormalities such as patella alta, mechanical impingement can be promoted.

MRI is the most useful imaging modality in the diagnosis of PFPIS.

In acute cases, MRI is useful to identify edematous and enlarged fat pads with nonencapsulated fibrous changes, demonstrated by a mass-like protrusion of fatty tissue into the suprapatellar pouch with low signal in T1-weighted imagers and high signal in fluid-sensitive sequences.

Fat pad edema and, furthermore, hyperemia can be evaluated by ultrasound, but the evaluation could be limited by the overlying suprapatellar bursa [1].

Conservative management, including ice, anti-inflammatory medication, and painful activities decrease could be the key to rule with the inflammation. If it doesn’t work, an arthroscopic evaluation should be considered.
